# MMHC-OCPR: Prediction of Platinum Response and Recurrence Risk in Ovarian Cancer with Multimodal Deep Learning

**DOI:** 10.3390/biomedicines14020348

**Published:** 2026-02-02

**Authors:** Enyu Tang, Haoming Xia, Zhenlong Yuan, Yuting Zhao, Shengnan Wang, Zhenbang Ye, Shangshu Gao, Ziqi Zhou, Yuxi Zhao, Jia Zeng, Nenan Lyu, Jing Zuo, Ning Li, Jianming Ying, Lingying Wu

**Affiliations:** 1 Department of Gynecology Oncology, National Cancer Center/National Clinical Research Center for Cancer/Cancer Hospital, Chinese Academy of Medical Sciences and Peking Union Medical College, Beijing 100021, China; b2023003101@pumc.edu.cn (E.T.); yzlbetablocker@gmail.com (Z.Y.); b2023003100@pumc.edu.cn (Y.Z.); 13840598176@163.com (S.W.); drzhaoyuxi@163.com (Y.Z.); zack.zeng@yahoo.com (J.Z.); lyunenan@cicams.ac.cn (N.L.); zuojing01894@163.com (J.Z.); liningnci@126.com (N.L.); 2 School of Clinical Medicine, Tsinghua University, Beijing 100084, China; haomingxia@hotmail.com; 3 Department of Pathology, National Cancer Center/National Clinical Research Center for Cancer/Cancer Hospital, Chinese Academy of Medical Sciences and Peking Union Medical College, Beijing 100021, Chinagss200076@163.com (S.G.); 4 State Key Laboratory of Power System Operation and Control, Department of Electrical Engineering, Tsinghua University, Beijing 100084, China; zhouzq23@mails.tsinghua.edu.cn

**Keywords:** ovarian cancer, MMHC-OCPR, clustering-constrained attention multiple instance learning, UNI2-h, platinum response, recurrence risk

## Abstract

**Background/Objectives**: Ovarian cancer has the highest mortality among gynecological malignancies, with platinum resistance significantly contributing to poor prognosis. We aimed to develop a multimodal model (MMHC-OCPR) to predict platinum response and recurrence risk, enabling earlier personalized treatment and improved outcomes. **Methods**: This multicenter retrospective study included a combined cohort of 431 patients, comprising 1182 whole slide images (WSIs) curated from two independent datasets. The primary cohort consisted of 376 patients from the National Cancer Center (China), which was further partitioned into training, validation and internal test sets to ensure model development and evaluation. An additional external test cohort was incorporated using publicly available data from TCGA, enhancing the generalizability of our findings. We implemented a weakly supervised multiple instance learning framework to integrate histopathological imaging with clinicopathological variables, further strengthened by the incorporation of the transformer-based pretrained encoder UNI2-h, which enhanced the model’s predictive performance. **Results**: All patients in the primary cohort had pathology slides collected from primary ovarian tumors and metastatic tumor, along with clinical factors related to prognosis and treatment response. The baseline platinum response classifier using primary WSIs achieved an AUC of 0.896 in the internal test group and 0.876 in the external test group. Integration of metastatic WSIs and clinical data inputs yielded a superior AUC of 0.914 in the internal test set. The recurrence risk model demonstrated a C-index of 0.801, rising to 0.838 after multimodal enhancement. The model stratified patients into low-, intermediate- and high-risk groups with 2-year progression-free survival rates of 77.3%, 48.0% and 2.0%, respectively. **Conclusions**: Our model enables the early detection of platinum resistance, guiding timely treatment intensification. The recurrence risk stratification supports personalized management by identifying patients with favorable outcomes following surgery and chemotherapy, potentially sparing them from maintenance therapy to reduce associated toxicity, cost, and enhance quality of life.

## 1. Introduction

Ovarian cancer remains the most lethal gynecologic malignancy [1]. Global statistics from 2020 reported 313,959 new cases of ovarian, fallopian tube, and primary peritoneal cancers, with 207,252 attributable deaths [2]. Approximately 70% of ovarian cancers are diagnosed at advanced stages. The conventional first-line treatment for advanced disease consists of primary or interval debulking surgery (PDS/IDS) followed by platinum-based combination chemotherapy, but median overall survival and progression-free survival (PFS) remain limited at approximately 30 and 12 months, respectively [3]. The incorporation of targeted agents such as bevacizumab and poly ADP-ribose polymerase inhibitors (PARPi) into first-line chemotherapy or maintenance therapy has significantly improved survival outcomes [4,5,6]. Emerging therapies including antibody-drug conjugates and immune checkpoint inhibitors show promise for platinum-resistant cases, defined as recurrence within 6 months post-chemotherapy [7,8,9].

Current first-line therapies face several clinical challenges: (1) Significant prognostic heterogeneity after platinum chemotherapy, particularly between platinum-sensitive and -resistant subgroups, coupled with a lack of reliable predictive biomarkers; (2) Potential selection bias in maintenance therapy due to the inclusion of platinum-sensitive patients who might benefit from chemotherapy alone [3]; (3) Substantial physiological and psychological burdens associated with maintenance agents like PARPi and bevacizumab, resulting from prolonged use and notable adverse effects [10,11]. These challenges highlight the urgent need for early predictive models to optimize personalized treatment and reduce toxicity.

Current biomarkers for platinum response, including BRCA1/2 mutations, HRD status, CA125, and imaging, demonstrate suboptimal predictive performance [12]. Histopathology encodes comprehensive disease information for diagnosis, classification, and prognostication. Although the chemotherapy response score (CRS) system enables pathologists to assess platinum response through residual tumor cell evaluation and fibroinflammatory changes [13], its clinical utility is limited by interobserver variability. Artificial intelligence (AI)-enhanced computational pathology improves the detection of subcellular and spatial features, using deep learning to identify prognostic patterns beyond human perception [14,15].

Recent advances in AI have extended its applications in pathology from diagnostic and molecular prediction to sophisticated outcome forecasting [16,17,18]. AI systems demonstrate growing capabilities in prognostic risk stratification and therapy response prediction through multimodal data integration [19,20]. Current models excel not only in baseline survival estimation but also in forecasting responses to chemotherapy, immunotherapy, and other novel treatments [21,22]. Tumors resulting from ovarian cancer metastasis to other sites may harbor a greater number of pathological features associated with invasiveness compared to the primary ovarian tumors, and these characteristics are closely linked to drug sensitivity and survival prognosis [23]. This study pioneers the integration of whole-slide image features from primary/metastatic lesions and clinicopathological variables using a weakly-supervised multiple instance learning framework (clustering-constrained attention multiple instance learning, CLAM) [24,25], building a multimodal model that predicts platinum response and recurrence risk in advanced ovarian cancer. As a clinical decision-support tool, it optimizes first-line therapy selection by enabling the early prediction of platinum resistance for timely treatment adjustment and stratifying recurrence risk to guide maintenance therapy—thus avoiding overtreatment, particularly in patients with excellent outcomes after surgery and platinum chemotherapy alone.

## 2. Materials and Methods

### 2.1. Patient Cohort

**Figure 1:**
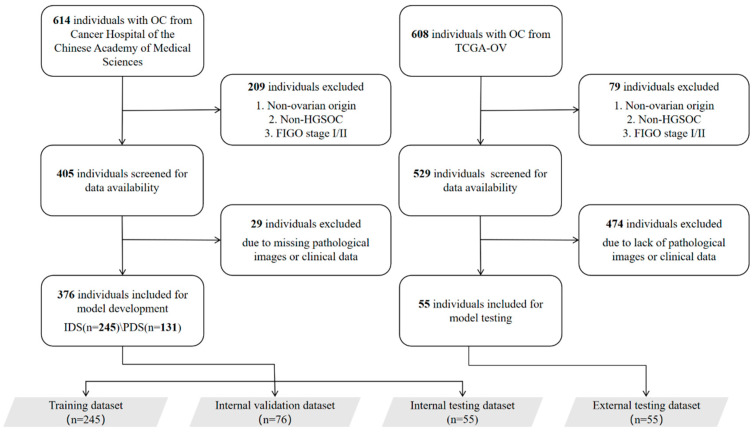
Workflow diagram for patient screening and dataset allocation. We screened 614 patients with ovarian malignant tumors from the National Cancer Center/Chinese Academy of Medical Sciences Cancer Hospital. After applying stringent inclusion and exclusion criteria, 376 patients were enrolled for model development and evaluation and assigned to the training, validation, and internal testing datasets. From the initial 608 patients in the TCGA-OV cohort, 55 met the same criteria and formed the external testing dataset for independent validation of the model’s predictive performance.

We retrospectively enrolled patients with advanced-stage (III–IV) high-grade serous ovarian carcinoma (HGSOC) treated at the National Cancer Center/Cancer Hospital, Chinese Academy of Medical Sciences between January 2010 and December 2015 (NCC dataset, Figure 1). Inclusion and exclusion criteria are described in Supplementary Note S1. This study was registered with the Chinese Clinical Trial Registry (date of the retrospective registration: 17 December 2025; Registration ID: ChiCTR2500114794; https://www.chictr.org.cn/bin/userProject (accessed on 30 September 2025)).

After rigorous screening, the NCC dataset, comprising 376 patients and containing a total of 1127 WSIs of H&E-stained tissue sections, was selected for model development (Supplementary Figure S1). This included 750 WSIs of primary ovarian cancer (with 374/376 patients contributing 2 WSIs each and 2/376 patients contributing 1 WSI each) and 377 WSIs of metastatic tumors (with 375/376 patients contributing 1 WSI each and 1/376 patients contributing 2 WSIs). All metastatic tumors originated from the peritoneum. All slides were derived from FFPE tissue blocks and were digitized using a digital pathology scanner (NanoZoomer, Hamamatsu Photonics K.K., Hamamatsu City, Japan). Patients from the NCC dataset were randomly allocated into training, validation, and internal test cohorts in a ratio of 0.65:0.2:0.15. To evaluate the model’s generalizability on external data, the TCGA-OV dataset from the TCGA database (https://portal.gdc.cancer.gov/ (accessed on 9 February 2025)) was employed as an external test cohort for external validation. This dataset consists of 55 FFPE-derived WSIs of primary ovarian cancer from 55 patients. The TCGA dataset provides only primary WSIs; therefore, external validation of multimodal models was limited to single-modality components, while models integrating metastatic WSIs and clinical variables were evaluated only through internal validation.

Patients in the NCC dataset received platinum-based adjuvant therapy (specific surgical and detailed chemotherapeutic regimens are provided in Supplementary Note S2). They underwent clinical and/or radiological examinations periodically for efficacy and recurrence assessment. If the relapse occurred ≥6 months after completing prior platinum-based chemotherapy, the disease was defined as platinum-sensitive, and otherwise as platinum-resistant. Disease recurrence was defined as the objective evidence of confirmed disease progression based on radiographic, histological, or biomarker criteria. PFS was defined as the time from completion of prior platinum-based chemotherapy to the first documented progression or death from any cause. The follow-up cutoff date for all patients was 24 May 2024. The primary endpoints of this study were the platinum response (platinum-sensitive/resistant) and PFS.

### 2.2. Construction of MMHC-OCPR

#### 2.2.1. Preprocessing of Pathological Images

The images from the National Cancer Center/Cancer Hospital, Chinese Academy of Medical Sciences and those from TCGA-OV were acquired using different scanning protocols. To mitigate the discrepancies introduced by varying scanning devices and protocols, we applied a standardized preprocessing pipeline: First, whole-slide images (WSIs) were converted to RGB, and a uniform size was enforced: image patches were resized to the target dimensions (224 × 224 pixels) using bicubic interpolation to ensure consistent input size. Subsequently, normalization was performed: pixel values were scaled from [0, 255] to [0, 1] and then standardized using the mean and standard deviation corresponding to the pretrained model. This normalization strategy maps images from different sources into a unified feature space, effectively reducing the impact of device and protocol variations on model performance and enhancing the model’s generalizability across multi-institutional data. The preprocessed images were then fed into a pretrained feature encoder (such as UNI or CONCH) to extract deep features, which were subsequently used within the multiple-instance learning framework for further analysis.

#### 2.2.2. Tile Extraction Parameters

During the tile extraction stage, a standardized pipeline was employed to ensure consistency and efficiency. All whole-slide images were processed at the highest resolution level, corresponding to the native scanning resolution under a 40× objective (approximately 0.25 μm/pixel). The multi-resolution pyramid structure of each WSI was automatically identified using the OpenSlide library, from which image tiles were extracted. The tile size was uniformly set to 256 × 256 pixels, corresponding to a tissue area of approximately 6.4 μm × 6.4 μm, with a default stride of 256 pixels (no overlap) to balance computational efficiency and feature coverage. Tissue masks were generated via an automated segmentation pipeline: images were first converted to HSV color space, and the saturation channel was extracted. Median filtering (kernel size mthresh = 7) was applied for noise reduction, followed by binarization using a fixed threshold (sthresh = 8) or Otsu’s adaptive thresholding. Morphological closing (close = 4) was then performed to connect fragmented regions. Artifact removal was implemented through multiple steps: white-area filtering excluded background regions based on a saturation threshold; black-area filtering removed over-stained regions based on an RGB mean threshold; and a four_pt strategy was used to inspect tissue mask coverage around the tile center, ensuring that tiles primarily resided within valid tissue regions.

#### 2.2.3. Stain Normalization and Color Enhancement Strategy

To enhance the model’s cross-institutional generalizability, we adopted a stain normalization strategy based on a pretrained encoder rather than traditional H&E-specific methods. This normalization approach maps images from different sources into a unified feature space. The advantage of this method lies in the robust feature representations learned by the pretrained model from diverse natural images, enabling it to adapt to color variations caused by different staining protocols and thereby reducing interference from inter-institutional differences. All slides were converted to RGB format and normalized as described above prior to training, ensuring consistency in feature space. Regarding color enhancement, no additional augmentation techniques (such as color jitter or contrast adjustment) were introduced during training in order to preserve the original biological information of tissue morphology and avoid introducing artificial artifacts that could interfere with model learning.

#### 2.2.4. Definition of Tumor Regions

Tumor regions were defined entirely through an automated pipeline, without requiring pathologists to manually delineate regions of interest (ROIs). We performed fully automatic tissue segmentation on each WSI to identify all tissue-containing regions, from which image tiles were extracted. This approach ensures that the model can leverage morphological information from the entire slide and is suitable for whole-slide analysis tasks. Automated segmentation not only improves efficiency but also avoids subjective bias inherent in manual annotation, making it highly compatible with weakly supervised learning frameworks.

#### 2.2.5. Handling of Multiple WSIs per Patient During Training

During training, although each patient may contribute multiple WSIs (e.g., from primary and metastatic sites), we addressed the issue of repeated measures using a slide-level strategy. Specifically, the dataset was constructed with WSIs as the unit rather than patients: for each individual WSI, the attention mechanism of the MMHC-OCPR model aggregated features from all of its internal tiles to generate a slide-level prediction. During the evaluation phase, patient-level information was used only for subsequent analysis (e.g., aggregating predictions from multiple WSIs via mean or median to derive a patient-level outcome). However, the training process itself did not employ patient-level aggregation, thereby avoiding the risk of data leakage. This design ensures that the model learns content-relevant features rather than being biased by patient frequency.

#### 2.2.6. Workflow of Model Construction

The construction of the MMHC-OCPR model comprises two stages. Stage 1 involves the selection of the optimal WSI encoder, and Stage 2 entails the performance evaluation of the model using multimodal and metastatic site data as input. Firstly, we individually evaluated various open-source, pre-trained histopathology image encoders (including CONCH [26], CTransPath [27], GigaPath [28], Phikon-v2 [29], ResNet50, UNI [30], and UNI2-h [31]) using primary ovarian cancer WSIs. This evaluation assessed their feature extraction and predictive capabilities for the platinum response classification and recurrence prediction tasks, aiming to identify the optimal histopathology image encoder for the subsequent model development.

Subsequently, we innovatively constructed the multimodal model, integrating histopathology images with clinical baseline data. We first utilized primary ovarian cancer WSIs to evaluate the performance advantage conferred by the multimodal architecture for both prediction tasks. Considering that each patient had paired primary and metastatic samples, we further integrated primary ovarian cancer WSIs with metastatic WSIs to assess the potential performance gain from incorporating metastatic WSIs. This process aimed to determine the optimal predictive input configuration for the MMHC-OCPR model.

#### 2.2.7. MMHC-OCPR Architecture

**Figure 2:**
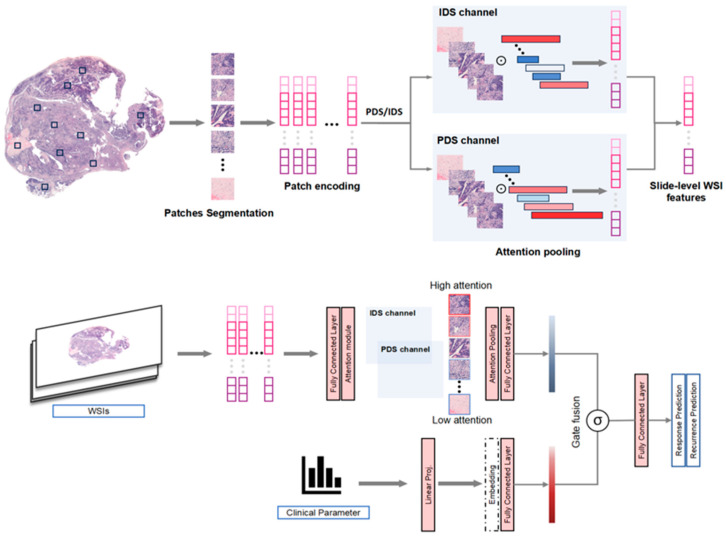
Architecture of MMHC-OCPR model. MMHC-OCPR is a deep learning model based on multi-modal attention fusion. It leverages a gated mechanism to dynamically integrate WSI features with clinical data, enabling the precise prediction of platinum response and recurrence risk in ovarian cancer patients. The model is designed to be applicable to patients undergoing either PDS and IDS. PDS, primary debulking surgery; IDS, internal debulking surgery.

Figure 2 presents an overview of the MMHC-OCPR model. This model extends the CLAM architecture [31] by innovatively integrating dual-modality data comprising histopathological images and clinical baseline information to enhance predictive performance for both platinum response classification and recurrence prediction tasks. Furthermore, the model incorporates dedicated attention networks for distinct surgery regimens (IDS/PDS). Each network employs a gated attention mechanism to discern the relative importance of individual image patches within the overall prediction task, generating representative image-level feature vectors via weighted aggregation. Subsequently, the model employs a gated fusion mechanism to jointly fuse WSI features and clinical features. This mechanism dynamically computes fusion weights based on the characteristics of both modalities, enabling adaptive balancing of the contributions from histopathological imaging and clinical data according to individual patient profiles and specific task requirements.

The integrated feature vector is fed into the output layer for final prediction. For the classification task, a dual-output structure is utilized to discriminate between different classes, optimized with a multi-objective loss function that combines bag-level cross-entropy loss and instance-level clustering loss to balance global and local feature learning. For the recurrence risk prediction task, a single-output structure generates continuous risk scores, with model optimization based on the Cox proportional hazards loss function. This framework not only fully leverages the spatial semantic information from WSIs and the clinical relevance of variables but also establishes an interpretable interaction mechanism between multimodal data. It provides a deep learning solution for personalized prognostic assessment and treatment decision-making in ovarian cancer.

We propose a multimodal attention-based deep learning framework that integrates WSI features with clinical data for predicting treatment response and recurrence in ovarian cancer. Our approach extends the CLAM architecture to handle dual-modality data through a gated fusion mechanism. Our model leverages several pretrained encoders, including CONCH, CTransPath, GigaPath, Phikon-v2, ResNet50, UNI, and UNI2-h, each specialized in extracting high-level features from WSIs. These encoders capture diverse tissue characteristics, enabling the effective integration of WSI features with clinical data through a gated fusion mechanism. The model employs two separate attention networks corresponding to different pre-treatment protocols (IDS or PDS), where each attention network follows a gated attention mechanism to compute attention weights $\alpha_{i}$ for each WSI patch. The weighted bag representation $M_{WSI}=\sum_{i=1}^{N}\alpha_{i}h_{i}$ (where $h_{i}$ represents the i-th patch feature and N is the total number of patches) is obtained through the weighted sum of patch features. To effectively integrate WSI features with clinical data, we designed a gated fusion mechanism that computes adaptive fusion weights $g=\sigma (f_{\mathrm{gate}}([M_{WSI};C])$ (where C denotes clinical features and σ is the sigmoid function) based on both modalities, allowing the model to dynamically balance the contribution of WSI and clinical features. The fused representation $M_{fused}$ is then passed through an output head to produce final predictions. The classification task employs a dual-output head, while the recurrence task uses a single-output head to generate the risk score.

The model employs distinct training configurations for classification and survival tasks. For classification task, a multi-objective loss function combines bag-level cross-entropy loss ($L_{bag}$) and instance-level clustering loss ($L_{inst}$): $L_{total}=L_{bag}+\lambda L_{inst}$ (λ = 0.9). Optimization uses the Adam optimizer (learning rate $5\times 10^{-5}$, weight decay $10^{-4}$), with training limited to 50 epochs and early stopping (patience 5). A dropout rate of 0.5 is applied to attention networks and fusion modules. For recurrence analysis, training leverages the Cox proportional hazards loss:

$$L=-\frac{1}{B}\sum_{i=1}^{B}\delta_{i}\left[{\hat{r}}_{i}-\log\sum_{j:t_{j}\geq t_{i}}\exp({\hat{r}}_{j})\right]$$

This incorporates event indicators ***δ***, survival times ***t***, and predicted risks $\hat{r}$. Optimization employs Adam (learning rate $2\times 10^{-4}$, weight decay $10^{-5}$) with a step scheduler, early stopping (patience 5), and dropout rate of 0.25 across feature extraction, clinical encoding, and survival layers. Both tasks use NVIDIA GeForce RTX 4080 GPU implementation, with fixed random seeds for reproducibility.

For each patient, the model processes multiple WSIs (including primary and metastatic lesions). After feature extraction, each WSI generates an independent prediction score (e.g., probability of platinum response or recurrence risk score). To consolidate this multi-slide information into a single patient-level prediction, we employed a statistical aggregation strategy. For classification tasks (such as platinum response prediction), we calculated the mean or median of the prediction scores across all WSIs to derive the final patient-level label. For survival analysis tasks (e.g., progression-free survival prediction), the risk scores from different WSIs were similarly aggregated by taking their mean or median.

#### 2.2.8. Heatmap Visualization

During the forward inference of the model, an attention module assigns an attention score to each patch, reflecting its contribution to the overall classification outcome. The features of all patches are then aggregated into a global representation through attention-weighted pooling, which is subsequently used for slide-level classification to produce the probability distribution across classes. Finally, the attention scores of individual patches are mapped back to their original spatial coordinates and overlaid onto the WSI to generate a heatmap, where the color intensity indicates the level of model attention.

### 2.3. Statistics

Quantitative variables are reported as median with interquartile range (IQR), and comparisons were made using the Kruskal–Wallis test, depending on the data distribution. Categorical variables were analyzed using the chi-square test or Fisher’s exact test. A *p*-value < 0.05 was considered statistically significant for all comparisons. The genomic profiling and functional enrichment analysis were performed utilizing R software (version 4.5.0) with the key packages including DESeq, clusterProfiler, xCell, and others. Model evaluation metrics included AUC or C-index with 95% CI. During the prediction model training phase, all models underwent 10 independent trials with different random seeds for validation and test set evaluations. For classification models, AUC served as the primary evaluation metric, with secondary metrics including precision, recall, F1-score, and specificity. Survival models were assessed using the C-index. The predictive performance across different models was compared based on the mean (95% CI) of the 10 training outcomes. Bootstrap resampling was employed to quantify the differences in the C-index and AUC and to generate corresponding two-sided *p*-values and 95% confidence intervals. This work has been reported in line with the STROCSS criteria [32].

## 3. Results

### 3.1. Baseline Characteristics

Baseline characteristics of the training, validation, and internal test sets derived from the NCC dataset are summarized in Table 1. The distribution of platinum-based chemotherapy response remained balanced across the groups, with platinum-sensitive patients accounting for 62.0% (152/245) in the training set, 61.8% (47/76) in the validation set, and 61.8% (34/55) in the internal test set. In the external TCGA-OV test set, platinum-sensitive patients constituted 72.7% (40/55).

Across the datasets, the median PFS was as follows: 8.8 months (95% CI: 7.6–10.7 months) in the training set, 9.6 months (95% CI: 8.0–17.6 months) in the validation set, 11.6 months (95% CI: 9.1–17.4 months) in the internal test set, and 14.0 months (95% CI: 12.0–17.6 months) in the external test set. The median PFS for the entire cohort was 10.2 months (95% CI: 9.1–11.7 months). The 1 year, 3 year, and 5 year PFS rates were 43.9% (95% CI: 39.4–48.8%), 13.6% (95% CI: 10.6–17.4%), and 7.1% (95% CI: 4.9–10.3%), respectively.

### 3.2. MMHC-OCPR: Development and Performance Evaluation of Platinum Response Prediction Model

To identify the optimal WSI feature encoder, we first evaluated the performance of pre-trained WSI encoders using primary ovarian cancer WSIs for both platinum-response prediction and recurrence prediction tasks. As shown in Supplementary Tables S1 and S2, the UNI2-h model demonstrated superior predictive performance in both tasks. For the platinum-response task, the AUC values in the training, validation, internal test, and external test sets were 0.956 (95% CI: 0.947–0.965), 0.909 (95% CI: 0.895–0.923), 0.884 (95% CI: 0.852–0.917), and 0.878 (95% CI: 0.839–0.917), respectively. For the recurrence prediction task, the C-index values were 0.836 (95% CI: 0.822–0.850), 0.782 (95% CI: 0.772–0.798), 0.762 (95% CI: 0.746–0.778), and 0.764 (95% CI: 0.751–0.777), respectively. Based on these results, UNI2-h was selected as the WSI encoder for subsequent development of the multimodal MMHC-OCPR model.

The MMHC-OCPR model integrates both WSI and baseline clinical data within a dual-modal framework and incorporates a dual-path architecture (IDS/PDS) to accommodate different treatment strategies. We compared the predictive performance of the multimodal MMHC-OCPR model with that of the unimodal UNI2-h pathology model for platinum-response classification in the NCC dataset. Given that each patient had two primary ovarian cancer WSIs, the prediction results from both slides were averaged to generate a patient-level prediction. As summarized in Table 2, MMHC-OCPR achieved higher AUC values than UNI2-h across all datasets: 0.967 (95% CI: 0.957–0.977) vs. 0.956 (95% CI: 0.947–0.965) in the training set, 0.929 (95% CI: 0.915–0.943) vs. 0.909 (95% CI: 0.895–0.923) in the validation set, and 0.903 (95% CI: 0.870–0.936) vs. 0.884 (95% CI: 0.852–0.917) in the internal test set, underscoring the performance advantage of the multimodal architecture.

Furthermore, we evaluated whether integrating primary and metastatic ovarian cancer WSIs within the pathological modality of the multimodal MMHC-OCPR model could enhance its performance in platinum-response prediction. As presented in Table 2 and the confusion matrix (Supplementary Figure S1), the multimodal MMHC-OCPR model incorporating both primary and metastatic WSIs demonstrated further improved predictive performance. The optimal results were achieved when multiple WSIs were aggregated at the patient level using median fusion, yielding AUC values of 0.960 (95% CI: 0.946–0.975) in the training set, 0.933 (95% CI: 0.920–0.946) in the validation set, and 0.912 (95% CI: 0.886–0.938) in the internal test set. Therefore, the multimodal MMHC-OCPR model, which integrates both primary and metastatic WSIs along with baseline clinical data, represents the optimal configuration for platinum-response prediction.

### 3.3. MMHC-OCPR: Development and Performance Evaluation for Recurrence Risk Prediction Model

To evaluate the PFS prediction capability of the MMHC-OCPR model, we compared the multimodal MMHC-OCPR model—which integrates primary ovarian cancer WSIs and baseline clinical data—against the unimodal UNI2-h pathology model in the NCC dataset. Predictions from two WSIs per patient were averaged to generate a patient-level risk score. As shown in Table 3, MMHC-OCPR demonstrated superior C-index performance compared to UNI2-h across all sets: training set, 0.868 (95% CI: 0.854–0.883) vs. 0.836 (95% CI: 0.822–0.850); validation set, 0.804 (95% CI: 0.794–0.814) vs. 0.782 (95% CI: 0.772–0.798); and internal test set, 0.793 (95% CI: 0.767–0.818) vs. 0.762 (95% CI: 0.746–0.778).

Subsequently, we incorporated metastatic WSIs into the MMHC-OCPR model. By aggregating predictions from multiple WSIs using median fusion to derive a patient-level risk score (designated as the MMHC-OCPR score), the model achieved optimal predictive performance (Table 3), with C-index values of 0.868 (95% CI: 0.852–0.883) in the training set, 0.822 (95% CI: 0.809–0.834) in the validation set, and 0.825 (95% CI: 0.796–0.854) in the internal test set. Thus, the multimodal MMHC-OCPR model integrating both primary and metastatic WSIs along with baseline clinical data represents the optimal configuration for PFS prediction.

We further assessed the prognostic value of the MMHC-OCPR score using Cox regression analysis with testing of Proportional Hazards (Supplementary Table S3). Univariate Cox regression analysis confirmed its significant association with PFS (Supplementary Table S4). Moreover, the MMHC-OCPR score remained an independent prognostic factor after adjusting for high-risk clinical variables, including high CA125 level, vascular tumor thrombus (VTT), FIGO stage IV, and suboptimal cytoreduction (Supplementary Table S5). To enhance clinical applicability and interpretability, patients were stratified into three risk groups—low-, intermediate-, and high-risk—based on tertile thresholds of the risk score derived from the training set. A progressive enrichment of high-risk clinical features was observed from the low-risk to the high-risk group (Supplementary Figure S2). Furthermore, the MMHC-OCPR risk groups retained independent prognostic value even after adjustment for clinical risk factors (Supplementary Table S6).

We subsequently evaluated the PFS predictive performance of the MMHC-OCPR risk groups. In the entire NCC dataset, the model achieved a C-index of 0.75 (95% CI: 0.73–0.77) and a 2 year time-dependent AUC of 0.87 (95% CI: 0.84–0.91). Across the training, validation, and internal test sets, the C-index values were 0.80 (95% CI: 0.78–0.81), 0.71 (95% CI: 0.66–0.76), and 0.74 (95% CI: 0.68–0.79), respectively, with corresponding 2 year time-dependent AUCs of 0.90 (95% CI: 0.87–0.93), 0.92 (95% CI: 0.85–0.99), and 0.88 (95% CI: 0.80–0.95).

**Figure 3:**
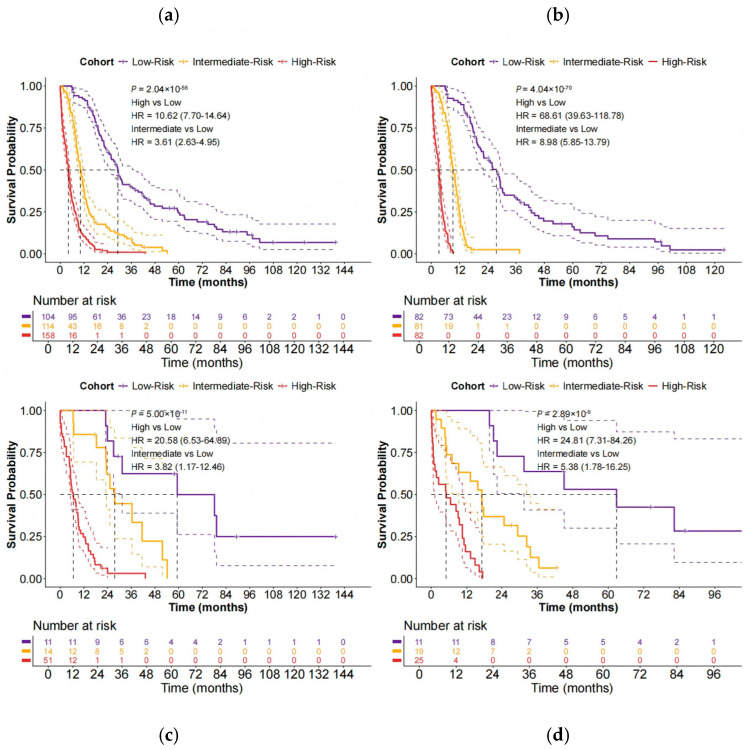
Kaplan–Meier curves of risk groups stratified by MMHC–OCPR. (**a**) NCC dataset. (**b**) Training dataset. (**c**) Validation dataset. (**d**) Internal testing dataset. Dashed lines indicated 95% CI.

Kaplan–Meier survival curves, shown in Figure 3, demonstrated clear prognostic stratification by the MMHC-OCPR groups across all datasets. Detailed survival data for patients stratified using the MMHC-OCPR model are provided in Supplementary Tables S5 and S7. In the entire NCC dataset (Supplementary Table S5), both median PFS and 2 year PFS rates exhibited a pronounced decreasing trend from the low-risk to the high-risk group: median PFS was 29.2, 10.2, and 4.1 months for the low-, intermediate-, and high-risk groups, respectively, and the corresponding 2 year PFS rates were 61.4%, 15.7%, and 1.0%. A consistent trend was observed in the training, validation, and internal test sets (Table 4), reflecting robust intergroup prognostic stratification.

Furthermore, to validate the model’s generalizability across different patient populations, we evaluated the performance of the MMHC-OCPR model within various clinical subgroups. As summarized in Supplementary Table S8, the model demonstrated great generalizability across diverse subpopulations.

### 3.4. Refined Risk Stratification Based on FIGO Staging

**Figure 4:**
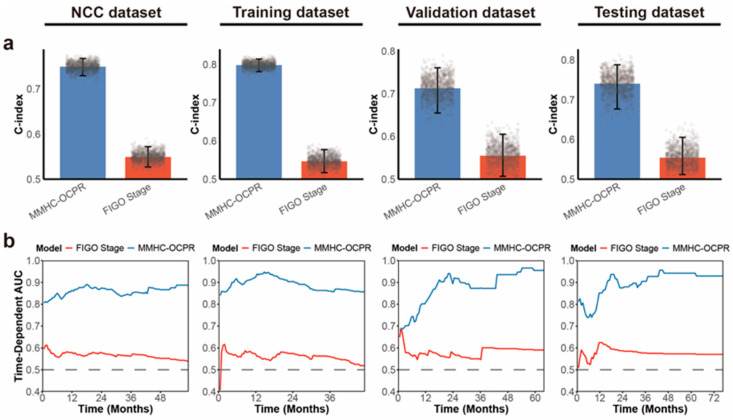
Comparison of PFS prediction performance between MMHC-OCPR and FIGO staging. This figure compares the predictive performance for PFS between the MMHC-OCPR model and the FIGO staging system, as assessed by the C-index (**a**) and time-dependent AUC (**b**) across the NCC, training, validation, and internal testing datasets.

To validate the clinical applicability of the MMHC-OCPR risk groups, we compared their prognostic performance with the guideline-recommended FIGO staging system (Figure 4 and Supplementary Table S9). The MMHC-OCPR model demonstrated significantly superior predictive performance across all datasets. To assess clinical utility, we compared decision curve analysis (DCA) curves of MMHC-OCPR and FIGO staging at the 1, 2, and 3 year time points. As shown in Supplementary Figure S3, the MMHC-OCPR model consistently provided the highest net benefit across all risk thresholds.

**Figure 5:**
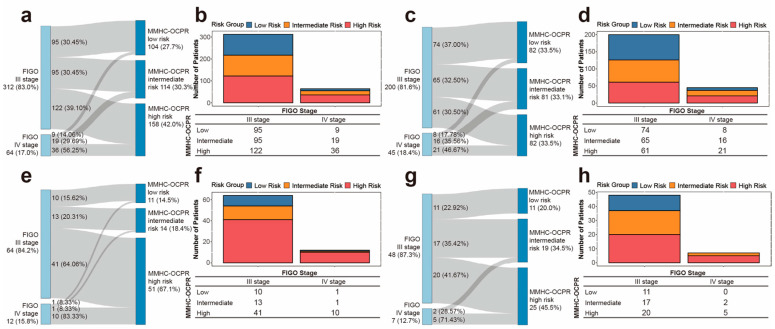
Risk restratification from FIGO staging to MMHC-OCPR risk groups. Sankey diagrams illustrate the restratification of patients from FIGO stages III–IV (left) to MMHC-OCPR risk groups (right) in the (**a**) NCC, (**c**) training, (**e**) validation, and (**g**) internal testing datasets. The corresponding bar charts and tables (**b**,**d**,**f**,**h**) display the detailed distribution of patients, color-coded by the MMHC-OCPR risk groups.

Furthermore, the MMHC-OCPR model enabled more refined risk stratification than FIGO staging. As illustrated in Figure 5, a substantial proportion of FIGO stage III patients (30.50–64.06%) across the datasets were up-classified into the MMHC-OCPR high-risk group, while 20.31–35.42% were reassigned to the intermediate-risk group. Conversely, among FIGO stage IV patients, 0–17.78% were down-classified to the low-risk group and 8.33–35.56% to the intermediate-risk group. Histograms further illustrated the quantitative redistribution of patients. In addition, compared with FIGO staging (Stage III: 2 year PFS rate 22.90–31.20%, median PFS 10.1–15.3 months; Stage IV: 2 year PFS rate 0–8.30%, median PFS 6.0–7.0 months; Supplementary Tables S10 and S11), the MMHC-OCPR risk groups exhibited more discriminative 2 year PFS rates and median PFS across all datasets (Table 4). To provide further support regarding model calibration and overall clinical reliability, Supplementary Tables S12–S14 show the calibration curves, Brier scores, and detailed censoring data distributions at key clinically relevant time points corresponding to the MMHC-OCPR model for each dataset.

### 3.5. Model Interpretation and Significant Features for the Prediction

**Figure 6:**
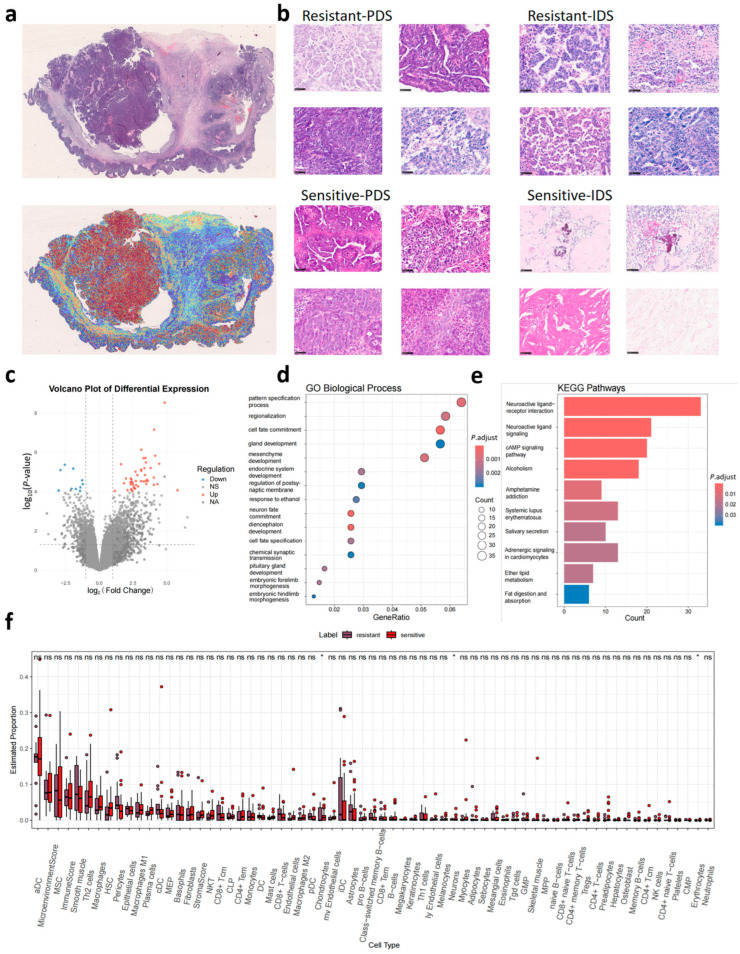
Model interpretation and significant features for the prediction model. (**a**) displays the whole slide image and prediction heatmap of a platinum-resistant patient. (**b**) presents the key discriminative pathological structures, as evaluated by expert pathologists, between the platinum-sensitive and platinum-resistant patients predicted by the model across PDS and IDS surgical types. (**c**) shows the differential gene expression volcano plot between predicted platinum-sensitive and resistant groups in the external test set. (**d**,**e**) shows the difference of enriched GO biological pathways (**d**), KEGG pathways (**e**), and cell clusters (**f**) between the predicted platinum-sensitive and resistant groups in the external testing dataset. PDS, primary debulking surgery; IDS, internal debulking surgery. * *p* < 0.05; ns: non-significant.

In collaboration with pathologists, we systematically analyzed histomorphological differences between the predicted platinum-sensitive and resistant groups using highly focused region heatmaps from decision-prediction classification from internal test set pathological images (Figure 6a). In PDS specimens (Figure 6b), both groups displayed solid and complex glandular patterns. However, resistant cases exhibited significantly higher proportions of these features, along with distinctive micropapillary architectures. In contrast, sensitive cases primarily showed papillary-solid patterns accompanied by tumor cell degeneration and neutrophil infiltration. For IDS cases, predicted resistant tumors maintained prominent micropapillary components and atypical tumor giant cells, whereas sensitive cases demonstrated treatment-related changes including psammomatous calcifications, sclerotic stroma, and edematous stroma with hyalinization.

Transcriptomic analysis of TCGA cases revealed significant pathway enrichment differences between the predicted groups. Differential gene expression analysis demonstrated significant enrichment in developmental processes through GO analysis, particularly in pattern specification processes, regionalization, and cell fate commitment. KEGG pathway analysis identified three key signaling pathways: neuroactive ligand–receptor interactions, neuroactive ligand signaling, and cAMP signaling pathway (Figure 6c–e). xCell deconvolution analysis showed no significant differences in immune cell infiltration between platinum-sensitive and resistant groups (Figure 6f), aligned with the well-established immune-cold tumor microenvironment in high-grade serous ovarian carcinoma.

## 4. Discussion

Ovarian cancer, the most challenging gynecologic malignancy to treat, often exhibits drug resistance as a key factor contributing to poor prognosis. Accurate prediction of therapeutic response at an early treatment stage is therefore crucial for timely treatment adjustment and personalized management. This study employed a systematic approach: first, we evaluated seven pre-trained encoder models using the CLAM framework, identifying UNI2-h as the optimal architecture for subsequent development. For platinum response prediction, a multi-phase optimization strategy significantly improved the model performance by progressively increasing input slide numbers (incorporating both primary ovarian and metastatic lesions) and integrating key clinical features as multimodal data. The optimized model achieved an AUC of 0.914 in internal testing, demonstrating notable generalizability. Furthermore, our recurrence risk prediction model successfully stratified patients into distinct risk groups, with significant survival differences (*p* < 0.001) consistently observed between low-, intermediate-, and high-risk groups across the validation and test sets, providing reliable guidance for clinical decision-making.

By integrating pathologists’ expert assessments with histomorphological analysis, we developed an interpretable predictive model. In PDS samples, the platinum-resistant group predicted by the model exhibited aggressive pathological features, including marked cellular atypia, high proportions of micropapillary and solid patterns—findings consistent with known poor prognostic markers [33]. In contrast, the platinum-sensitive group showed tumor cell degeneration and neutrophil infiltration, suggesting enhanced chemotherapy efficacy through inflammatory responses [34]. For IDS samples after adjuvant chemotherapy, the model demonstrated similar discriminative power. The predicted platinum-resistant group retained micropapillary dominance and atypical tumor giant cells, while the platinum-sensitive group exhibited treatment-responsive features such as psammoma calcifications, stromal sclerosis, and hyalinized edematous stroma—pathological changes indicative of therapeutic efficacy [13,35]. GO analysis revealed key differentiated enriched pathways, including the pattern specification process, regionalization, and cell fate commitment, in platinum-sensitive vs. resistant recurrent cases, implicating disrupted spatial organization and differentiation in tumor initiation and heterogeneity [36,37]. Furthermore, KEGG analysis identified neuroactive ligand–receptor interaction and cAMP signaling pathways as potential drivers of proliferation, metastasis, and treatment resistance [38,39,40].

Employing the CLAM architecture with attention-based learning, our model identified diagnostically critical subregions in WSIs and refined feature representation through instance-level clustering [30]. UNI2-h is an enhanced version of UNI, a large-scale vision transformer (ViT-Large) pretrained on more than 100 million pathology images. This self-supervised model outperforms existing methods in computational pathology, supporting cross-resolution classification, few-shot learning, and cancer subtype generalization, thereby advancing AI applications in diverse clinical diagnostics [31,41]. By integrating metastatic tumor tissue and key clinical factors, we developed a highly accurate predictive model for platinum response and recurrence risk in ovarian cancer. This facilitates early intervention in platinum-resistant cases and identifies best-prognosis patients who may safely avoid maintenance therapy, thereby reducing treatment-related toxicity and psychological burden.

Unlike prior models that primarily rely on primary tumor pathology or limited clinical variables [24,25,42,43], our approach uniquely integrates whole-slide images from both primary and metastatic lesions, capturing the tumor’s spatial heterogeneity. Recognizing that ovarian cancer metastases might exhibit distinct pathological features associated with aggressiveness compared to the primary tumor [23], we have validated that incorporating whole-slide images from metastatic lesions further improves the predictive accuracy of our model beyond that achieved using primary tumor pathology alone. Furthermore, it incorporates key intraoperative and pathological determinants such as cytoreductive surgery (CRS) score and completeness of cytoreduction, which are strongly prognostic but often omitted in purely image-based models. This multimodal integration of dual-site pathology and granular clinical data provides a more comprehensive biological and clinical profile, which likely contributes to the notable performance observed in our test set. Our work thus proposes a more holistic framework for prognostication, moving beyond conventional single-source models.

This study has several limitations. First, the model was developed exclusively on a cohort from China, while external validation employed a more heterogeneous population from the United States with differing demographic and clinical characteristics. Although the model demonstrated notable performance, this geographical and clinical disparity may affect its generalizability to other healthcare settings. Furthermore, the external validation cohort comprised only 55 patients. Therefore, the relatively large hazard ratios (HRs) observed in the Kaplan–Meier analysis should be interpreted with consideration for the limited sample size. Future multi-center, multinational studies are warranted to further validate and calibrate the model across diverse populations. Second, our model incorporates only histopathological and clinical data; including additional modalities such as genomic or radiomic features could further enhance predictive performance. Finally, as maintenance therapy has become standard in advanced ovarian cancer, extending our model to predict responses to maintenance therapy could refine personalized treatment strategies and better identify patients who are most likely to benefit.

## 5. Conclusions

In summary, this study developed an advanced AI model that integrates histopathological images and clinical data to accurately predict treatment outcomes in ovarian cancer patients after cytoreductive surgery and platinum-based chemotherapy. By leveraging a reinforced multimodal architecture based on CLAM and the pre-trained encoder UNI-2h, the model achieved high predictive accuracy through the deep integration of heterogeneous data modalities, yielding clinically actionable insights. The model serves as an effective clinical decision-support tool. It optimizes frontline therapeutic strategy by enabling the early prediction of platinum resistance and the stratification of recurrence risk, thus facilitating timely treatment modifications and informed maintenance therapy decisions.

## Figures and Tables

**Table 1:** Clinical characteristics of the training, validation, and internal testing datasets.

Characteristics	Training	Validation	Internal Testing	*p*
	(*N* = 245)	(*N* = 76)	(*N* = 55)	
Age (year), median (IQR)	56.0 [49.0; 61.0]	56.5 [48.0; 60.0]	54.0 [47.0; 62.5]	0.884
FIGO stage, *n* (%)				0.573
III	200 (81.6%)	64 (84.2%)	48 (87.3%)	
IV	45 (18.4%)	12 (15.8%)	7 (12.7%)	
Optimal cytoreduction, *n* (%)				0.362
Optimal	79 (32.2%)	19 (25.0%)	14 (25.5%)	
Suboptimal	166 (67.8%)	57 (75.0%)	41 (74.5%)	
VTT, *n* (%)				0.291
Negative	205 (83.7%)	59 (77.6%)	42 (76.4%)	
Positive	40 (16.3%)	17 (22.4%)	13 (23.6%)	
CRS, *n* (%)				0.053
1	69 (28.2%)	15 (19.7%)	13 (23.6%)	
2	92 (37.6%)	19 (25.0%)	17 (30.9%)	
3	13 (5.3%)	4 (5.26%)	3 (5.5%)	
N (PDS)	71 (29.0%)	38 (50.0%)	22 (40.0%)	
Lymph resection, *n* (%)				0.101
None	111 (45.3%)	24 (31.6%)	30 (54.5%)	
Enlarged node resection	35 (14.3%)	13 (17.1%)	5 (9.09%)	
Systematic resection	99 (40.4%)	39 (51.3%)	20 (36.4%)	
Diabetes, *n* (%)				0.678
No	232 (94.7%)	72 (94.7%)	54 (98.2%)	
Yes	13 (5.31%)	4 (5.26%)	1 (1.82%)	
Hypertension, *n* (%)				0.584
No	192 (78.4%)	58 (76.3%)	46 (83.6%)	
Yes	53 (21.6%)	18 (23.7%)	9 (16.4%)	
CA12-5 (U/mL), median (IQR)	1188 [472; 2871]	1044 [472; 3040]	1233 [427; 2104]	0.810
Menopause, *n* (%)				0.788
No	158 (64.5%)	47 (61.8%)	33 (60.0%)	
Yes	87 (35.5%)	29 (38.2%)	22 (40.0%)	
Menopause age, median (IQR)	49.0 [46.0; 51.0]	50.0 [47.8; 52.0]	49.0 [46.0; 50.5]	0.124
Height (cm), median (IQR)	158 [155; 161]	158 [154; 161]	158 [155; 161]	0.555
Weight (kg), median (IQR)	59.0 [54.0; 65.0]	59.0 [54.8; 63.2]	58.0 [52.5; 63.0]	0.877
BMI (kg/m^2^), median (IQR)	23.5 [21.5; 26.0]	23.6 [21.9; 25.9]	23.4 [21.6; 24.8]	0.528
No. of cycles, median (IQR)				
Neoadjuvant cycles	2.00 [0.00; 3.00]	0.50 [0.00; 2.00]	1.00 [0.00; 2.00]	0.002
Post surgical cycles	6.00 [5.00; 6.00]	6.00 [5.75; 6.00]	6.00 [5.00; 6.00]	0.126
Total cycles	8.00 [7.00; 8.00]	7.50 [6.00; 8.00]	7.00 [6.00; 8.00]	0.014
Surgery type, *n* (%)				0.352
PDS	71 (29.0%)	38 (50.0%)	22 (40.0%)	
IDS	174 (71.0%)	38 (50.0%)	33 (60.0%)	
Platinum response, *n* (%)				0.999
Resistant	93 (38.0%)	29 (38.2%)	21 (38.2%)	
Sensitive	152 (62.0%)	47 (61.8%)	34 (61.8%)	

Descriptive data for categorical variables are reported as counts (percentages), and for continuous variables as medians with IQR, respectively. IQR, interquartile range; VTT, vascular tumor thrombus; CRS, chemotherapy response score.

**Table 2:** Comparative performance of models for platinum-response prediction.

Model	AUC	Precision	Recall	F1	Specificity
UNI2-h (Unimodal)					
Training	0.956 (0.947, 0.965)	0.866 (0.843, 0.889)	0.816 (0.773, 0.859)	0.820 (0.761, 0.879)	0.778(0.704, 0.852)
Validation	0.909 (0.895, 0.923)	0.734 (0.696, 0.773)	0.775 (0.755, 0.795)	0.775 (0.745, 0.804)	0.733 (0.694, 0.772)
Internal Testing	0.884 (0.852, 0.917)	0.745 (0.637, 0.854)	0.787 (0.745, 0.828)	0.772 (0.697, 0.848)	0.738 (0.631, 0.845)
MMHC-OCPR (Multimodal)					
Training	0.967 (0.957, 0.977)	0.872 (0.820, 0.923)	0.890 (0.827, 0.953)	0.863 (0.786, 0.940)	0.784 (0.656, 0.912)
Validation	0.929 (0.915, 0.943)	0.808 (0.784, 0.831)	0.820 (0.792, 0.847)	0.789 (0.757, 0.822)	0.698 (0.640, 0.756)
Internal Testing	0.903 (0.870, 0.936)	0.774 (0.708, 0.840)	0.773 (0.691, 0.854)	0.733 (0.634, 0.832)	0.612 (0.444, 0.780)
MMHC-OCPR (+Metastatic WSI/Mean)					
Training	0.922 (0.888, 0.955)	0.798 (0.754, 0.843)	0.797 (0.705, 0.889)	0.756 (0.628, 0.884)	0.643 (0.423, 0.864)
Validation	0.893 (0.873, 0.914)	0.758 (0.741, 0.775)	0.738 (0.707, 0.769)	0.684 (0.639, 0.728)	0.521 (0.437, 0.605)
Internal Testing	0.871 (0.848, 0.893)	0.740 (0.717, 0.762)	0.711 (0.656, 0.766)	0.652 (0.559, 0.745)	0.488 (0.294, 0.683)
MMHC-OCPR (+Metastatic WSI/Median)					
Training	0.960 (0.946, 0.975)	0.839 (0.778, 0.899)	0.847 (0.774, 0.921)	0.810 (0.719, 0.900)	0.695 (0.547, 0.842)
Validation	0.933 (0.920, 0.946)	0.805 (0.784, 0.826)	0.804 (0.775, 0.833)	0.762 (0.723, 0.801)	0.632 (0.555, 0.709)
Internal Testing	0.912 (0.886, 0.938)	0.782 (0.762, 0.802)	0.776 (0.751, 0.802)	0.733 (0.692, 0.773)	0.600 (0.483, 0.717)

This table compares the performance of different model input configurations across the training, validation, and internal testing datasets. The UNI2-h model utilizes unimodal whole-slide image (WSI) data from primary ovarian cancer sites only. In contrast, the MMHC-OCPR (Multimodal) model integrates WSIs from primary sites with baseline clinical data. Two advanced versions, MMHC-OCPR (+Metastatic WSI/Mean) and MMHC-OCPR (+Metastatic WSI/Median), further incorporate WSIs from metastatic lesions, with all tiles aggregated at the patient level by mean and median fusion, respectively.

**Table 3:** Comparative Performance of Models in Predicting Recurrence Risk.

Models	C-Index		
	Train	Validation	Internal Testing
UNI2-h (Unimodal)	0.836 (0.822, 0.850)	0.782 (0.772, 0.798)	0.762 (0.746, 0.778)
MMHC-OCPR (Multimodal)	0.868 (0.854, 0.883)	0.804 (0.794, 0.814)	0.793 (0.767, 0.818)
MMHC-OCPR (+Metastatic WSI/Mean)	0.863 (0.838, 0.887)	0.814 (0.800, 0.828)	0.793 (0.763, 0.823)
MMHC-OCPR (+Metastatic WSI/Median)	0.868 (0.852, 0.883)	0.822 (0.809, 0.834)	0.825 (0.796, 0.854)

This table compares the performance of different model input configurations across the training, validation, and internal testing datasets. The UNI2-h model utilizes unimodal whole-slide image (WSI) data from primary ovarian cancer sites only. In contrast, the MMHC-OCPR (Multimodal) model integrates WSIs from primary sites with baseline clinical data. Two advanced versions, MMHC-OCPR (+Metastatic WSI/Mean) and MMHC-OCPR (+Metastatic WSI/Median), further incorporate WSIs from metastatic lesions, with all tiles aggregated at the patient level by mean and median fusion, respectively.

**Table 4:** Patient distribution and PFS outcomes by risk group stratified by MMHC-OCPR.

MMHC-OCPR	Training Dataset			Validation Dataset			Internal Testing Dataset		
	Low Risk (N = 82)	Intermediate Risk (N = 81)	High Risk (N = 82)	Low Risk (N = 11)	Intermediate Risk (N = 14)	High Risk (N = 51)	Low Risk (N = 11)	Intermediate Risk (N = 19)	High Risk (N = 25)
No. of recurrence	69	79	82	7	11	49	7	17	25
2-yr PFS rate	57.10%	2.50%	0.00%	81.80%	62.30%	3.10%	72.70%	36.80%	0.00%
Median PFS (months)	27.7 (22.0–30.4)	9.2 (8.2–10.7)	3.3 (2.8–4.1)	59.3 (31.4–NA)	27.6 (23.1–NA)	6.6 (5.2–9.2)	62.8 (31.5–NA)	17.1 (9.3–33.7)	5.0 (1.3–10.5)

Progression-free survival (PFS) is presented as median with 95% CI. NA: Due to the limited sample size, the upper confidence limit of the survival curve remained above a 50% survival rate throughout the entire observation period; therefore, the upper bound estimate for this interval could not be determined.

## Data Availability

The datasets from the Cancer Hospital, Chinese Academy of Medical Sciences are subject to access restrictions as they were used in this study with participant consent. Anonymized data may be made available to qualified researchers upon formal request to the corresponding authors. The H&E-stained digital WSIs from the public TCGA-OV dataset were obtained via the National Cancer Institute Genomic Data Commons Portal (https://portal.gdc.cancer.gov/). The codebase is publicly accessible at https://github.com/TEY-ops/clam_oc.

## Supplementary Materials

The following supporting information can be downloaded at: https://www.mdpi.com/article/10.3390/biomedicines14020348/s1. Supplementary note: Note S1. Inclusion criteria and exclusion criteria; Note S2. Therapy regimens; Supplementary figures: Figure S1. Confusion matrix of the MMHC-OCPR (+Metastatic WSI/Median); Figure S2. Distribution of clinical risk characteristics based on MMHC-OCPR stratification; Figure S3. Decision curve analysis for different staging models at one-, two-, and three-years; Supplementary tables: Table S1. Comparing the performance of different encoders in the training, validation, internal testing, and external testing datasets in predicting platinum response; Table S2. Comparing the performance of different encoders in the training, validation, internal testing, and external testing datasets in predicting recurrence risk; Table S3. Testing of proportional hazards for Cox regression analysis; Table S4. Univariate Cox regression analysis MMHC-OCPR and clinical risk factors; Table S5. Multivariate Cox regression analysis of MMHC-OCPR score and clinical risk factors; Table S6. Multivariate Cox regression analysis of MMHC-OCPR groups and clinical risk factors; Table S7. Patient distribution and PFS outcomes by risk group stratified by MMHC-OCPR; Table S8. Performance comparison of MMHC-OCPR in predicting PFS across different subgroups; Table S9. Comparative analysis of PFS prediction performance between MMHC-OCPR and FIGO staging; Table S10. Patient distribution and PFS outcomes by risk group stratified by FIGO staging in the NCC dataset; Table S11. Patient distribution and PFS outcomes by risk group stratified by FIGO staging in the training, validation, and internal testing datasets; Table S12. Calibration curves corresponding to the MMHC-OCPR model for each dataset; Table S13. Brier scores corresponding to the MMHC-OCPR model for each dataset; Table S14. Detailed censoring data distributions at key clinically relevant time points corresponding to the MMHC-OCPR model for each dataset.

## Abbreviations

The following abbreviations are used in this manuscript:

WSI	Whole Slide Image
AUC	Area Under Curve
PDS	Primary Debulking Surgery
IDS	Interval Debulking Surgery
PARPi	Poly ADP-Ribose Polymerase Inhibitors
CRS	Chemotherapy Response Score
AI	Artificial Intelligence
CLAM	Clustering-Constrained Attention Multiple Instance Learning
HGSOC	High-Grade Serous Ovarian Carcinoma
FFPE	Formalin-Fixed, Paraffin-Embedded
PFS	Progression Free Survival
IQR	Interquartile Range
CI	Confidence Interval
VTT	Vascular Tumor Thrombus
