# Supplementary material for: MMHC-OCPR: Prediction of Platinum Response and Recurrence Risk in Ovarian Cancer with Multimodal Deep Learning
Source: Biomedicines. 2026 Feb 2;14(2):348. doi: 10.3390/biomedicines14020348 (PMC12938349; doi:10.3390/biomedicines14020348)
Supplement: Supplementary file 1 [file biomedicines-14-00348-s001.zip › biomedicines-4087549-supplementary.pdf]

Table of contents

METHODS

Note 1. Inclusion criteria and exclusion criteria

Note 2. Therapy Regimens

FIGURES

Fig. 1. Confusion matrix of the MMHC-OCPR (+Metastatic WSI /Median)

Fig. 2. Distribution of clinical risk characteristics based on MMHC-OCPR stratification

Fig. 3. Decision curve analysis for different staging models at one-, two-, and three-years

TABLES

Table 1. Comparing the performance of different encoders in the training, validation, internal testing and external testing datasets in predicting platinum response

Table 2. Comparing the performance of different encoders in the training, validation, internal testing and external testing datasets in predicting recurrence risk

Table 3. Testing of Proportional Hazards for Cox regression analysis

Table 4. Univariate Cox regression analysis MMHC-OCPR and clinical risk factors

Table 5. Multivariate Cox regression analysis of MMHC-OCPR score and clinical risk factors

Table 6. Multivariate Cox regression analysis of MMHC-OCPR groups and clinical risk factors

Table 7. Patient distribution and PFS outcomes by risk group stratified by MMHC-OCPR

Table 8. Performance comparison of MMHC-OCPR in predicting PFS across different subgroups

Table 9. Comparative analysis of PFS prediction performance between MMHC-OCPR and FIGO staging

Table 10. Patient distribution and PFS outcomes by risk group stratified by FIGO staging in the NCC dataset

Table 11. Patient distribution and PFS outcomes by risk group stratified by FIGO staging in the training, validation and internal testing datasets

Table 12. Calibration curves corresponding to the MMHC-OCPR model for each dataset

Table 13. Brier scores corresponding to the MMHC-OCPR model for each dataset

Table 14. Detailed censoring data distributions at key clinically relevant time points corresponding to the MMHC-OCPR model for each dataset

Supplementary Note 1

Inclusion criteria include:

1) Completion of 6-8 cycles of platinum-based neoadjuvant or adjuvant chemotherapy; 2) Availability of tumor tissue slides confirmed as HGSOC after PDS/IDS;

3) Documented clinical outcomes including disease progression and other clinical information;

4) No prior exposure to maintenance therapy before first recurrence;

5) Absence of concurrent primary malignancies at other sites.

Exclusion criteria include:

1) Non-ovarian primary cancer;

2) Lack of or necessary clinical data or pathological slides of the primary and metastatic tumors;

3) prior exposure to maintenance therapy before first recurrence;

4) Presence of concurrent primary malignancies at other sites.

Supplementary Note 2

Therapy Regimens

If the patient is assessed as having a high likelihood of optimal cytoreduction during initial evaluation, primary cytoreductive surgery is performed. Otherwise, the patient receives 2–3 cycles of neoadjuvant chemotherapy, after which they are re-evaluated for the feasibility of optimal cytoreduction before undergoing interval cytoreductive surgery. The cytoreductive procedure includes hysterectomy with bilateral salpingo-oophorectomy, omentectomy, and resection of metastatic lesions, with or without pelvic and para-aortic lymphadenectomy. The chemotherapy regimen consists of a platinum-based agent combined with paclitaxel administered every 3 to 4 weeks per cycle, with a total of 6–8 cycles of neoadjuvant and adjuvant chemotherapy.

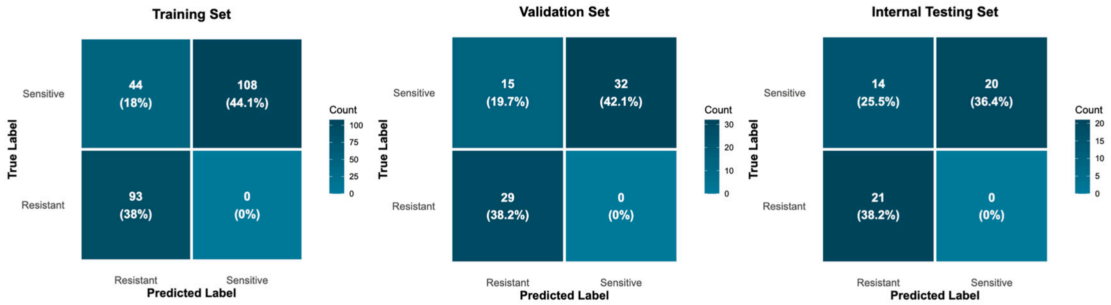

Fig. 1 Confusion matrix of the MMHC-OCPR (+Metastatic WSI /Median)

Given that all model training in this study was performed with five independent runs using different random seeds for training and evaluation, we selected the experimental results from the run with the best performance on the internal validation set during the training of the MMHC-OCPR (+Metastatic WSI / Median) model and plotted the confusion matrices for the training set, validation set, and internal test set.

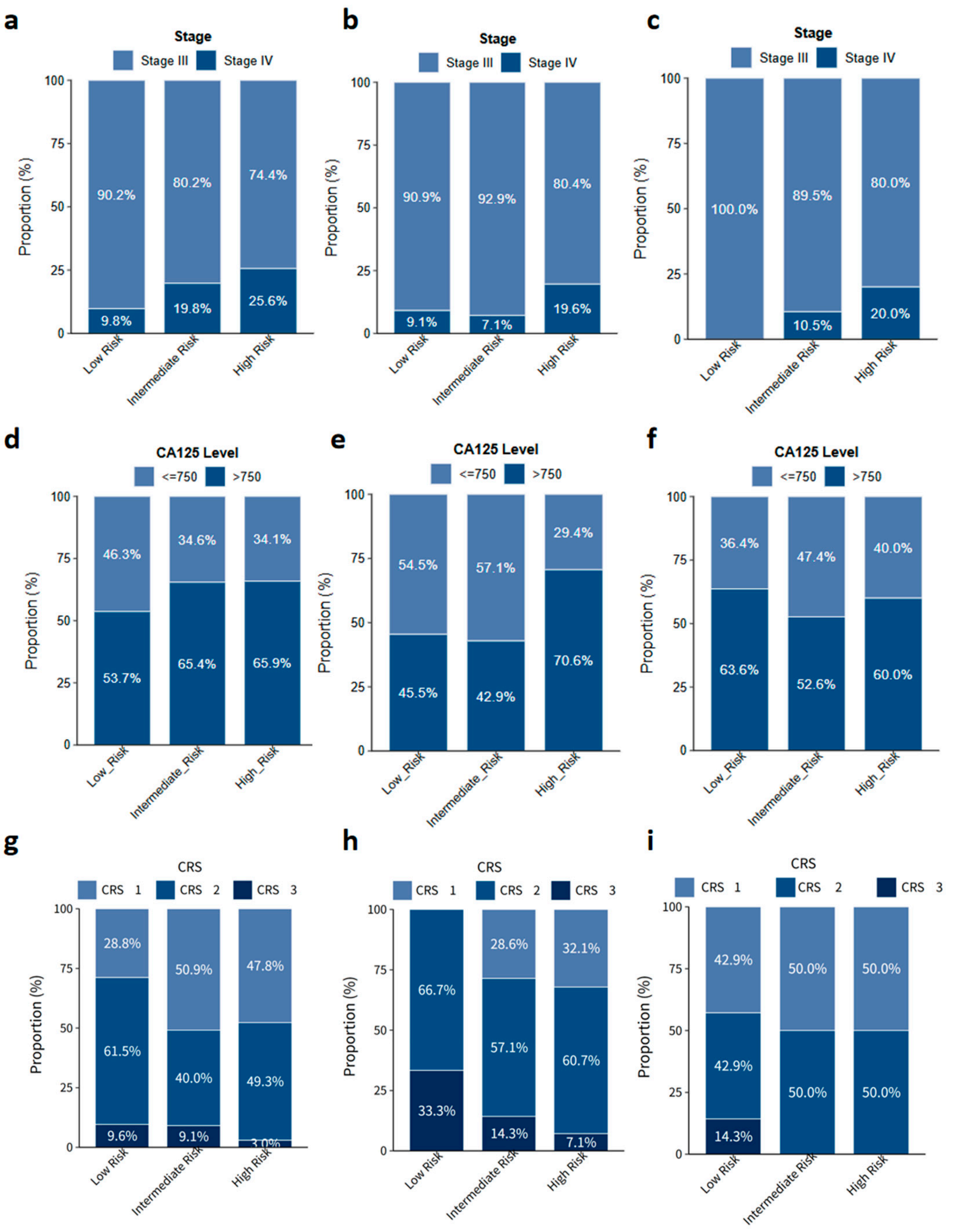

Fig. 2 Distribution of clinical risk characteristics based on MMHC-OCPR stratification

This figure illustrates the distribution of clinical characteristics across MMHC-OCPR risk groups in the training (a, d, g), validation (b, e, h), and internal testing (c, f, i) sets. The analysis revealed a progressive enrichment of FIGO stage IV cases from the low-risk to the high-risk group (training set: 25.6%; validation set: 19.6%; testing set: 20.0%). Furthermore, the high-risk group demonstrated a higher distribution of elevated CA125 levels. Additionally, the distribution of preoperative treatment response types was consistent across the risk groups, with Response 3 being most prevalent in the low-risk group and least prevalent in the high-risk group.

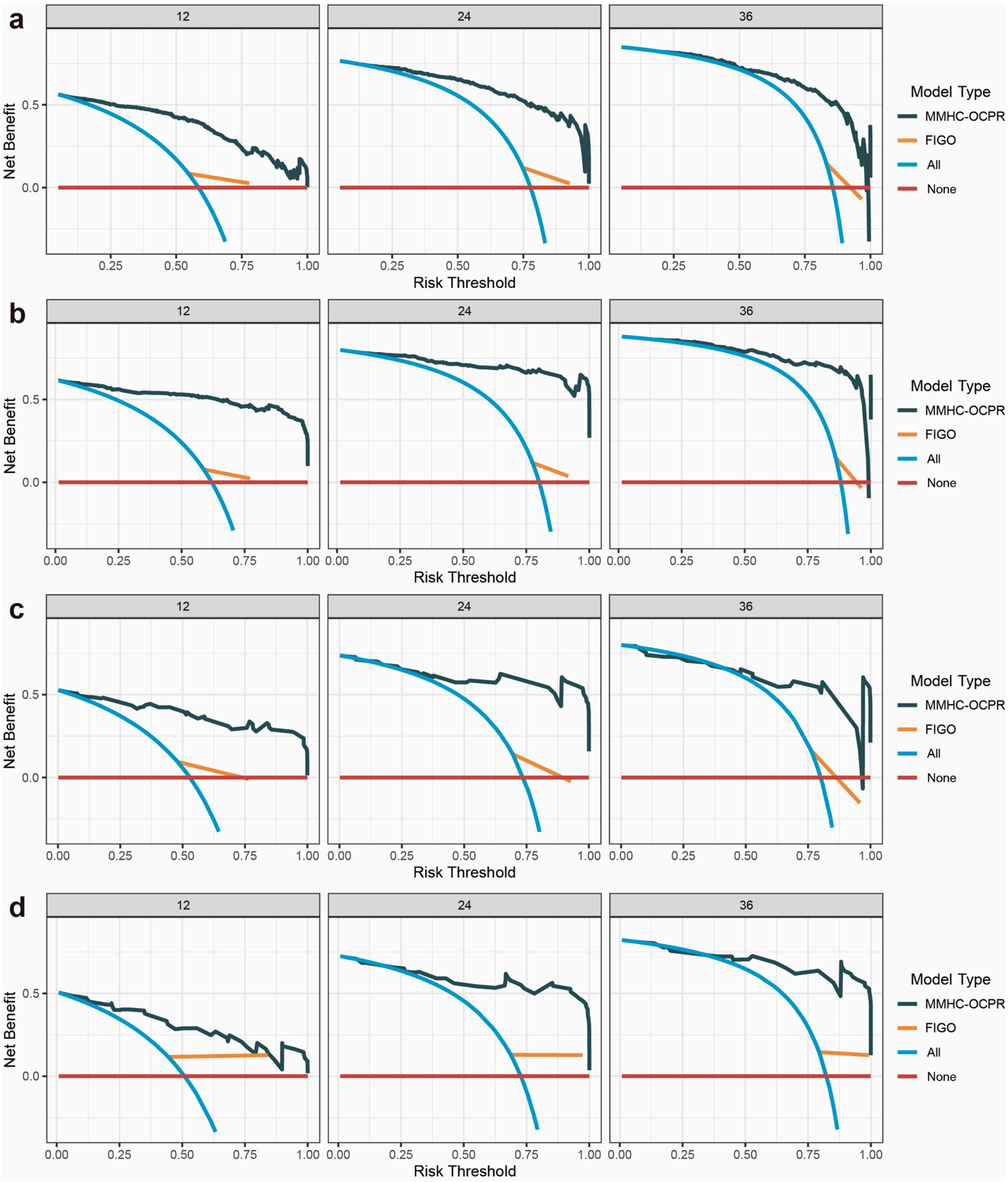

Fig. 3 Decision curve analysis for different staging models at one-, two-, and three-years

Panels (a), (b), (c) and (d) show DCA curves for the NCC, training, validation, and internal testing datasets, respectively. Each panel compares the net benefit of different prognostic models (MMHC-OCPR and FIGO) for predicting 1-, 2-, and 3-year PFS. The y-axis indicates net benefit, and the x-axis indicates risk threshold. “All” and “None” represent strategies of treating all or no patients.

Table 1 Comparing the performance of different encoders in the training, validation, internal testing and external testing datasets in predicting platinum response

Model/Dataset	AUC	Precision	Recall	F1	Specificity
**CONCH**					
Training	0.900 (0.875, 0.925)	0.813 (0.766, 0.860)	0.775 (0.750, 0.800)	0.770 (0.727, 0.813)	0.722 (0.665, 0.780)
Validation	0.847 (0.828, 0.865)	0.684 (0.644, 0.724)	0.716 (0.691, 0.742)	0.718 (0.686, 0.750)	0.683 (0.643, 0.722)
Internal Testing	0.813 (0.768, 0.859)	0.644 (0.589, 0.698)	0.684 (0.669, 0.699)	0.679 (0.650, 0.708)	0.640 (0.586, 0.695)
External Testing	0.823 (0.762, 0.884)	0.607 (0.525, 0.690)	0.662 (0.623, 0.700)	0.684 (0.639, 0.730)	0.594 (0.527, 0.660)
**CTransPath**					
Training	0.911 (0.902, 0.919)	0.833 (0.814, 0.851)	0.782 (0.776, 0.789)	0.783 (0.769, 0.797)	0.739 (0.719, 0.759)
Validation	0.850 (0.835, 0.865)	0.676 (0.634, 0.719)	0.718 (0.697, 0.739)	0.715 (0.684, 0.745)	0.674 (0.632, 0.716)
Internal Testing	0.865 (0.842, 0.888)	0.658 (0.612, 0.704)	0.703 (0.678, 0.728)	0.700 (0.664, 0.736)	0.657 (0.610, 0.705)
External Testing	0.830 (0.784, 0.876)	0.655 (0.468, 0.841)	0.720 (0.624, 0.815)	0.687 (0.656, 0.719)	0.621 (0.491, 0.750)
**GigaPath**					
Training	0.910 (0.890, 0.929)	0.819 (0.775, 0.863)	0.783 (0.755, 0.811)	0.776 (0.729, 0.823)	0.727 (0.665, 0.789)
Validation	0.867 (0.851, 0.884)	0.664 (0.627, 0.702)	0.732 (0.709, 0.756)	0.716 (0.686, 0.746)	0.662 (0.623, 0.701)
Internal Testing	0.847 (0.816, 0.877)	0.600 (0.534, 0.666)	0.681 (0.644, 0.718)	0.658 (0.605, 0.711)	0.595 (0.526, 0.665)
External Testing	0.833 (0.773, 0.892)	0.553 (0.444, 0.662)	0.653 (0.626, 0.679)	0.663 (0.611, 0.715)	0.548 (0.452, 0.644)
**Phikon-v2**					
Training	0.948 (0.929, 0.967)	0.798 (0.730, 0.867)	0.804 (0.749, 0.858)	0.795 (0.707, 0.883)	0.746 (0.633, 0.859)
Validation	0.884 (0.868, 0.899)	0.703 (0.658, 0.748)	0.746 (0.717, 0.774)	0.743 (0.707, 0.779)	0.701 (0.656, 0.746)
Internal Testing	0.855 (0.825, 0.886)	0.655 (0.561, 0.748)	0.709 (0.672, 0.746)	0.697 (0.635, 0.759)	0.650 (0.556, 0.745)
External Testing	0.853 (0.793, 0.914)	0.589 (0.457, 0.721)	0.668 (0.628, 0.708)	0.684 (0.623, 0.745)	0.581 (0.467, 0.695)
**ResNet50**					
Training	0.876 (0.861, 0.890)	0.758 (0.717, 0.798)	0.754 (0.746, 0.763)	0.754 (0.734, 0.775)	0.713 (0.678, 0.748)
Validation	0.838 (0.817, 0.860)	0.664 (0.627, 0.702)	0.708 (0.690, 0.726)	0.703 (0.678, 0.728)	0.662 (0.626, 0.698)
Internal Testing	0.802 (0.745, 0.859)	0.662 (0.575, 0.749)	0.707 (0.671, 0.742)	0.699 (0.643, 0.755)	0.658 (0.572, 0.743)
External Testing	0.830 (0.776, 0.884)	0.691 (0.581, 0.800)	0.704 (0.669, 0.739)	0.742 (0.690, 0.793)	0.672 (0.581, 0.764)
**UNI**					

Training	0.935 (0.922, 0.948)	0.829 (0.773, 0.885)	0.801 (0.788, 0.815)	0.807 (0.781, 0.833)	0.766 (0.729, 0.803)
Validation	0.874 (0.858, 0.889)	0.696 (0.663, 0.729)	0.742 (0.725, 0.760)	0.738 (0.714, 0.762)	0.695 (0.661, 0.728)
Internal Testing	0.878 (0.853, 0.902)	0.676 (0.598, 0.755)	0.752 (0.720, 0.783)	0.729 (0.674, 0.784)	0.673 (0.592, 0.754)
External Testing	0.857 (0.798, 0.916)	0.582 (0.502, 0.662)	0.679 (0.663, 0.694)	0.696 (0.657, 0.734)	0.578 (0.503, 0.652)
**UNI2-h**					
Training	0.956 (0.947, 0.965)	0.866 (0.843, 0.889)	0.816 (0.773, 0.859)	0.820 (0.761, 0.879)	0.778 (0.704, 0.852)
Validation	0.909 (0.895, 0.923)	0.734 (0.696, 0.773)	0.775 (0.755, 0.795)	0.775 (0.745, 0.804)	0.733 (0.694, 0.772)
Internal Testing	0.884 (0.852, 0.917)	0.745 (0.637, 0.854)	0.787 (0.745, 0.828)	0.772 (0.697, 0.848)	0.738 (0.631, 0.845)
External Testing	0.878 (0.839, 0.917)	0.644 (0.490, 0.797)	0.713 (0.638, 0.788)	0.730 (0.662, 0.798)	0.634 (0.500, 0.768)

Table 2 Comparing the performance of different encoders in the training, validation, internal testing and external testing datasets in predicting recurrence risk

**Models**	**C-Index**			
	**Train**	**Validation**	**Internal Testing**	**External Testing**
CONCH	0.766 (0.750, 0.782)	0.713 (0.694, 0.733)	0.727 (0.708, 0.746)	0.707 (0.684, 0.729)
CTransPath	0.831 (0.817, 0.845)	0.750 (0.737, 0.762)	0.738 (0.716, 0.761)	0.745 (0.728, 0.762)
GigaPath	0.808 (0.793, 0.823)	0.737 (0.721, 0.752)	0.737 (0.718, 0.757)	0.704 (0.691, 0.717)
Phikon-v2	0.821 (0.800, 0.842)	0.755 (0.731, 0.779)	0.726 (0.710, 0.743)	0.737 (0.713, 0.761)
ResNet50	0.760 (0.739, 0.781)	0.706 (0.684, 0.729)	0.683 (0.654, 0.712)	0.725 (0.709, 0.740)
UNI	0.801 (0.785, 0.817)	0.736 (0.720, 0.753)	0.753 (0.724, 0.782)	0.714 (0.701, 0.726)
UNI2-h	0.836 (0.822, 0.850)	0.782 (0.772, 0.798)	0.762 (0.746, 0.778)	0.764 (0.751, 0.777)

Table 3 Testing of Proportional Hazards for Cox regression analysis

Variable	Train dataset		Validation dataset		Internal testing dataset	
	**Chisq**	***P***	**Chisq**	***P***	**Chisq**	***P***
**MMHC-OCPR score**	0.125	0.724	3.193	0.055	0.5321	0.47
**CA12-5: >750**	2.476	0.116	0.461	0.497	2.6459	0.10
**VTT**	4.189	0.073	0.740	0.390	0.6905	0.41
**FIGO stage: IV**	0.122	0.727	0.745	0.388	0.9622	0.33
**Suboptimal cytoreduction**	0.121	0.728	0.144	0.704	0.0421	0.84
**GLOBAL**	8.223	0.144	6.255	0.282	5.7580	0.33

Variable	Train dataset		Validation dataset		Internal testing dataset	
	**Chisq**	***P***	**Chisq**	***P***	**Chisq**	***P***
**MMHC-OCPR group**	3.28062	0.194	3.0801	0.21	1.5535	0.46
**CA12-5: >750**	2.11468	0.146	0.5898	0.44	1.0629	0.30
**VTT**	4.65788	0.051	2.4036	0.12	0.9592	0.33
**FIGO stage: IV**	0.00994	0.921	0.6159	0.43	1.5585	0.21
**Suboptimal cytoreduction**	2.37657	0.123	0.0211	0.88	0.0321	0.86
**GLOBAL**	5.97448	0.50	5.2435	0.51	5.8955	0.43

Table 4 Univariate Cox regression analysis MMHC-OCPR and clinical risk factors

Variable	Entire (NCC) dataset		Train dataset		Validation dataset		Internal testing dataset	
	HR (95%CI)	*P*	HR (95%CI)	*P*	HR (95%CI)	*P*	HR (95%CI)	*P*
**MMHC-OCPR score**	1.64 (1.55-1.73)	**<0.0001**	2.42 (2.19-2.66)	**<0.0001**	2.24 (1.82-2.74)	**<0.0001**	2.17 (1.70-2.75)	**<0.0001**
**MMHC-OCPR: Intermediate risk**	3.61 (2.63-4.95)	**<0.0001**	8.98 (5.85-13.79)	**<0.0001**	3.82 (1.17-12.46)	**0.0263**	5.38 (1.78-16.25)	**0.0029**
**MMHC-OCPR: High risk**	10.62 (7.70-14.64)	**<0.0001**	68.61 (39.63-118.78)	**<0.0001**	20.58 (6.53-64.89)	**<0.0001**	24.81 (7.31-84.26)	**<0.0001**
**CA12-5: >750**	1.27 (1.02-1.58)	**0.0314**	1.25 (0.96-1.63)	0.1017	1.65 (1.00-2.74)	0.0504	0.92 (0.52-1.62)	0.7664
**VTT**	1.23 (0.94-1.60)	0.1287	1.10 (0.78-1.56)	0.5804	1.32 (0.76-2.31)	0.3211	1.73 (0.89-3.35)	0.1042
**FIGO stage: IV**	1.91 (1.45-2.51)	**<0.0001**	1.67 (1.20-2.31)	**0.0023**	2.21 (1.17-4.19)	**0.0146**	3.13 (1.32-7.42)	**0.0096**
**Suboptimal cytoreduction**	1.61 (1.27-2.02)	**0.0001**	1.43 (1.08-1.88)	**0.0125**	2.14 (1.21-3.81)	**0.0093**	1.71 (0.91-3.22)	0.0969

Results are presented as HR with 95% CI for Univariate Cox analyses. A two-sided P value <0.05 was considered statistically significant.

Bold entries depict statistical significance.

Table 5 Multivariate Cox regression analysis of MMHC-OCPR score and clinical risk factors

**Variable**	**Entire (NCC) dataset**		**Train dataset**		**Validation dataset**		**Internal testing dataset**	
	**HR (95%CI)**	***P***	**HR (95%CI)**	***P***	**HR (95%CI)**	***P***	**HR (95%CI)**	***P***
**MMHC-OCPR score**	1.65 (1.56-1.74)	**<0.0001**	2.50 (2.25-2.77)	**<0.0001**	2.18 (1.78-2.67)	**<0.0001**	2.18 (1.70-2.79)	**<0.0001**
**CA12-5: >750**	1.23 (0.99-1.53)	0.0594	1.42 (1.08-1.88)	**0.0124**	1.33 (0.77-2.30)	0.3079	1.13 (0.59-2.17)	0.7118
**VTT**	0.91 (0.69-1.19)	0.4753	0.72 (0.50-1.03)	0.0716	0.92 (0.52-1.63)	0.7816	1.47 (0.72-3.02)	0.2911
**FIGO stage: IV**	1.63 (1.23-2.16)	**0.0007**	1.17 (0.83-1.64)	0.3726	1.53 (0.78-3.02)	0.2180	1.79 (0.70-4.61)	0.2247
**Suboptimal cytoreduction**	1.70 (1.34-2.15)	**<0.0001**	1.17 (0.88-1.56)	0.2702	2.12 (1.11-4.05)	**0.0224**	1.45 (0.73-2.88)	0.2920

Results are presented as HR with 95% CI for multivariable analyses. A two-sided P value <0.05 was considered statistically significant.

Bold entries depict statistical significance.

Table 6 Multivariate Cox regression analysis of MMHC-OCPR groups and clinical risk factors

**Variable**	**Entire (NCC) dataset**		**Train dataset**		**Validation dataset**		**Internal testing dataset**	
	**HR (95%CI)**	***P***	**HR (95%CI)**	***P***	**HR (95%CI)**	***P***	**HR (95%CI)**	***P***
**MMHC-OCPR: Intermediate risk**	3.46 (2.52-4.76)	**<0.0001**	9.40 (6.05-14.61)	**<0.0001**	3.16 (1.01-10.34)	**0.0416**	4.95 (1.56-15.72)	**0.0067**
**MMHC-OCPR: High risk**	10.72 (7.73-14.85)	**<0.0001**	73.82 (41.83-130.28)	**<0.0001**	17.97 (5.51-58.58)	**<0.0001**	25.56 (7.23-90.40)	**<0.0001**
**CA125: >750**	1.22 (0.98-1.52)	0.0709	1.30 (0.99-1.71)	0.0619	1.34 (0.78-2.32)	0.2875	0.96 (0.51-1.79)	0.8898
**VTT**	1.07 (0.82-1.40)	0.6302	0.88 (0.62-1.25)	0.4807	1.11 (0.61-1.99)	0.735	1.99 (0.94-4.22)	0.0706
**FIGO stage: IV**	1.84 (1.39-2.43)	**<0.0001**	1.50 (1.07-2.09)	**0.0189**	2.24 (1.13-4.43)	**0.0209**	1.91 (0.75-4.87)	0.1783
**Suboptimal cytoreduction**	1.67 (1.31-2.11)	**<0.0001**	1.25 (0.94-1.67)	0.1232	2.46 (1.34-4.53)	**0.0036**	1.34 (0.64-2.83)	0.4352

Results are presented as HR with 95% CI for multivariable analyses. A two-sided P value <0.05 was considered statistically significant.

Bold entries depict statistical significance. VTT, vascular tumor thrombus.

Table 7 Patient distribution and PFS outcomes by risk group stratified by MMHC-OCPR groups in NCC dataset

**MMHC-OCPR**	**NCC dataset**		
	Low risk (N = 104)	Intermediate risk (N = 114)	High risk (N = 158)
No. of recurrence	83	107	156
2-yr PFS rate	61.40%	15.70%	1.00%
Median PFS (months)	29.2 (25.7-37.7)	10.2 (9.1-11.8)	4.1 (3.4-5.0)

Progression-free survival (PFS) is presented as median with 95% CI.

Table 8 Performance comparison of MMHC-OCPR in predicting PFS across different subgroups

Subgroups	Numbers	C-index	*P*	2-year AUC	*P*
**Age**					
≤60 years	272	0.75 (0.73-0.77)	Reference	0.88 (0.84-0.92)	Reference
>60 years	104	0.75 (0.71-0.79)	1	0.85 (0.79-0.92)	0.534
**VTT**					
Absent	306	0.75 (0.73-0.77)	Reference	0.87 (0.83-0.91)	Reference
Present	70	0.74 (0.69-0.79)	0.688	0.89 (0.82-0.96)	0.686
**CA12-5**					
≤750	146	0.75 (0.72-0.78)	Reference	0.87 (0.82-0.92)	Reference
>750	230	0.75 (0.72-0.78)	1	0.87 (0.82-0.92)	1
**Pausimenia**					
Absent	238	0.75 (0.73-0.77)	Reference	0.86 (0.82-0.91)	Reference
Present	138	0.76 (0.73-0.79)	0.69	0.89 (0.84-0.94)	0.55
**Treatment**					
PDS	131	0.74 (0.70-0.78)	Reference	0.85 (0.79-0.92)	Reference
IDS	245	0.75 (0.73-0.77)	0.676	0.89 (0.85-0.93)	0.4
**FIGO stage**					
III	312	0.75 (0.73-0.77)	Reference	0.87 (0.83-0.91)	Reference
IV	64	0.72 (0.66-0.78)	0.424	0.90 (0.80-1.00)	0.721

Numbers in parentheses are 95% confidence intervals. A two-sided P value <0.05 was considered statistically significant.

Table 9 Comparative analysis of PFS prediction performance between MMHC-OCPR and FIGO staging

Datasets	Models	C-index	*P*	2-year AUC	*P*
Entire (NCC) dataset	MMHC-OCPR	0.75 (95%CI: 0.73-0.77)	Reference	0.87 (95%CI: 0.84-0.91)	Reference
	FIGO	0.55 (95%CI: 0.53-0.57)	**<0.0001**	0.58 (95%CI: 0.54-0.61)	**<0.0001**
Training dataset	MMHC-OCPR	0.80 (95%CI: 0.78-0.81)	Reference	0.90 (95%CI: 0.87-0.93)	Reference
	FIGO	0.55 (95%CI: 0.52-0.58)	**<0.0001**	0.57 (95%CI: 0.53-0.62)	**<0.0001**
Validation dataset	MMHC-OCPR	0.71 (95%CI: 0.66-0.76)	Reference	0.92 (95%CI: 0.85-0.99)	Reference
	FIGO	0.56 (95%CI: 0.51-0.61)	**<0.0001**	0.57 (95%CI: 0.50-0.65)	**<0.0001**
Internal testing dataset	MMHC-OCPR	0.74 (95%CI: 0.68-0.79)	Reference	0.88 (95%CI: 0.80-0.95)	Reference
	FIGO	0.55 (95%CI: 0.51-0.61)	**<0.0001**	0.59 (95%CI: 0.53-0.65)	**<0.0001**

Numbers in parentheses are 95% confidence intervals.

Table 10 Patient distribution and PFS outcomes by risk group stratified by FIGO staging in the NCC dataset

FIGO stage	NCC dataset	
	III stage (N = 312)	IV stage (N = 64)
No. of recurrence	282	64
2-yr PFS rate	25.60%	6.20%
Median PFS (months)	10.7 (9.2-12.5)	6.2 (4.1-8.1)

Progression-free survival (PFS) is presented as median with 95% CI.

Table 11 Patient distribution and PFS outcomes by risk group stratified by FIGO staging in the training, validation and internal testing datasets

FIGO stage	Training dataset		Validation dataset		Internal testing dataset	
	III stage (N = 200)	IV stage (N = 45)	III stage (N = 64)	IV stage (N = 12)	III stage (N = 48)	IV stage (N = 7)
No. of recurrence	185	45	55	12	42	7
2-yr PFS rate	22.90%	6.70%	29.70%	8.30%	31.20%	0.00%
Median PFS (months)	10.1 (8.2-12.0)	6.0 (4.1-8.2)	12.7 (8.4-23.1)	6.2 (1.1-NA)	15.3 (9.5-21.0)	7.0 (0.9-NA)

Progression-free survival (PFS) is presented as median with 95% CI.

Table 12. Calibration curves corresponding to the MMHC-OCPR model for each dataset

NCC dataset

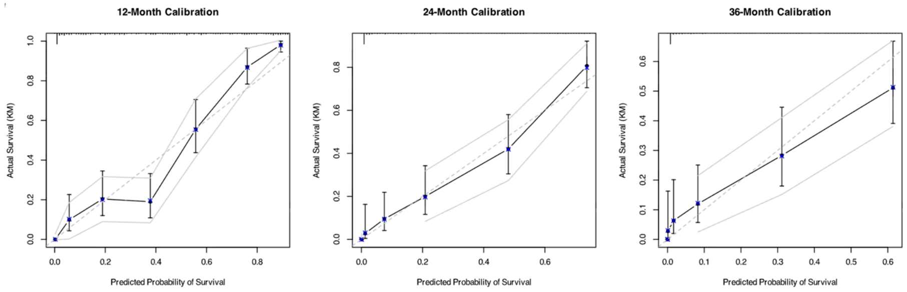

Training dataset

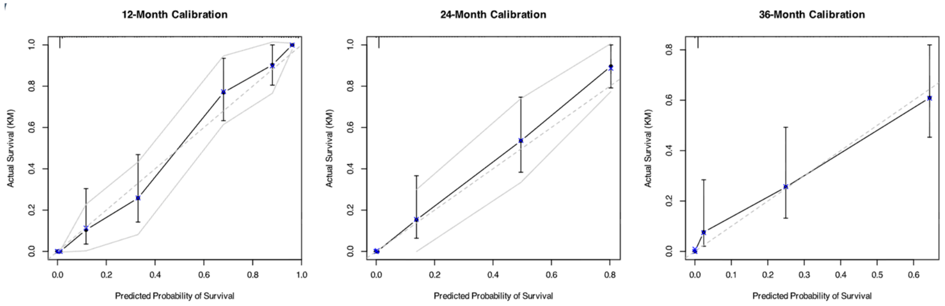

Validation dataset

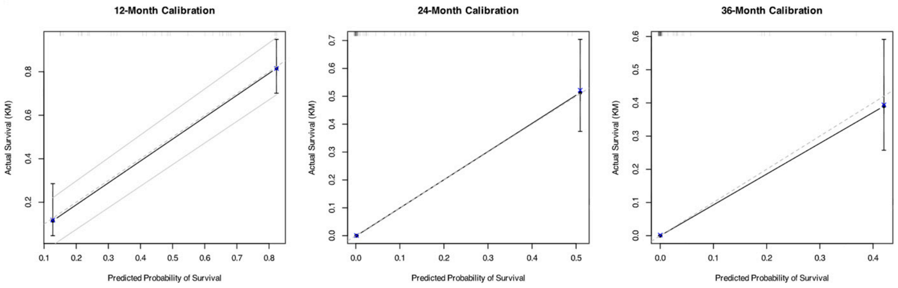

Internal testing dataset

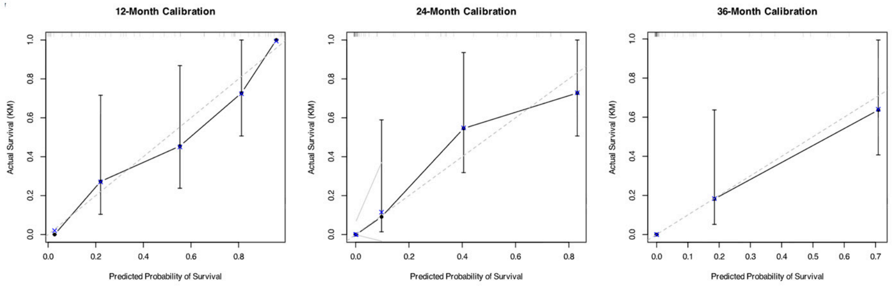

Table 13. Brier scores corresponding to the MMHC-OCPR model for each dataset

Time points	Brier scores		
	Training	Validation	Internal testing
1 year	0.070 (95%CI: 0.051-0.090)	0.099 (95%CI: 0.058-0.140)	0.136 (95%CI: 0.079-0.193)
2 years	0.059 (95%CI: 0.041-0.077)	0.093 (95%CI: 0.046-0.141)	0.100 (95%CI: 0.050-0.150)
3 years	0.058 (95%CI: 0.037-0.078)	0.120 (95%CI: 0.061-0.180)	0.082 (95%CI: 0.031-0.132)

Table 14. Detailed censoring data distributions at key clinically relevant time points corresponding to the MMHC-OCPR model for each dataset

Training dataset

Time Point	Number at Risk	Cumulative Events	Cumulative Censored	PFS Probability
1 year	92	151	2	0.381 (95%CI: 0.324-0.447)
2 years	45	194	6	0.198 (95%CI: 0.154-0.256)
3 years	24	211	10	0.119 (95%CI: 0.083-0.169)
5 years	9	223	13	0.055 (95%CI: 0.031-0.097)

Validation dataset

Time Point	Number at Risk	Cumulative Events	Cumulative Censored	PFS Probability
1 year	35	40	1	0.471 (95%CI: 0.371-0.599)
2 years	18	55	3	0.263 (95%CI: 0.179-0.386)
3 years	12	59	5	0.200 (95%CI: 0.125-0.320)
5 years	4	65	7	0.087 (95%CI: 0.038-0.202)

Internal testing dataset

Time Point	Number at Risk	Cumulative Events	Cumulative Censored	PFS Probability
1 year	27	28	0	0.491 (95%CI: 0.375-0.643)
2 years	15	40	0	0.273 (95%CI: 0.177-0.420)
3 years	9	45	1	0.176 (95%CI: 0.099-0.315)
5 years	5	47	3	0.131 (95%CI: 0.064-0.268)
